# Fictional faces of female suicide: Qualitative analysis of selected Russian-language texts of the school reader

**DOI:** 10.1192/j.eurpsy.2024.300

**Published:** 2024-08-27

**Authors:** E. B. Lyubov, N. D. Semenova

**Affiliations:** Moscow Research Institute of Psychiatry – a branch of the V. Serbsky National Medical Research Centre for Psychiatry and Narcology of the Ministry of Health of the Russian Federation, Moscow, Russian Federation

## Abstract

**Introduction:**

Isaiah Berlin’s (1948) exploration of the self-searching of Russian thinkers includes studies of the writers – Tolstoy and others (now – Russian-language texts of the school reader). These studies refute a widespread misconception about the relations between Russian writers and thinkers: namely, that in Russia literature and radical thought form two distinct traditions related only by mutual hostility. The works of Tolstoy, Dostoevsky, Karamzin, Leskov, Ostrovsky, and of minor novelists too, are penetrated with a sense of their own time, of this or that particular social and historical milieu and its ideological content, to an even higher degree than the ‘social’ novels of the west. The personal characteristics of suicide victims, heroines of Russian literature, along with the gender aspects, deserve attention in suicidal behavior (SP) focus.

**Objectives:**

To study personal characteristics of suicide victims, heroines of fiction.

**Methods:**

Qualitative analysis of selected Russian-language texts of the school reader.

**Results:**

At the dawn of literature, we have seen fiery heroines and tremulous victims in the arms of death. Аs psychology approach was developed, and we get acquainted with the tragiс backstory. Psychotypes of suicides are exaggerated and overlapped. «Hysterical»: manipulative, frigid nymphomaniac (e.g., Anna Karenina). «Freedom-loving rebel» (i.e., Katerina reincarnations from A. Ostrovsky) in conditions of excessive regulation. «She-Devil, or Rebel Without a Cause». The obsession with death turns into a criminal and a victim (e.g., «Lady Macbeth of the Mtsensk» by N. Leskov, Turgenev’s Susanna or Klara Milich). The image of a vindictively furious («velvet and tiger claws») woman descends to the Victorian view of female self-will. «Mimosa» is a sensitive, dreamy person, unable to resist the hardships of life, dependent (dies with her beloved, objection). In suicide, the strength of weakness is the outcome of a humiliating life (e.g., Karamzin’s «Poor Liza», «A Gentle Creature» by F. Dostoevsky. The meaning of suicides is the following: a call (to compassion, salvation), a «cry for help», atonement for one’s (imaginary) – someone else’s (by proxy) guilt, and release from encumbrance.

**Conclusions:**

Fiction and life are united by emotionally unstable characters and/or depression as markers of unsatisfactory resilience. The cultural diversity of gender patterns and interpretations of SP challenges the essentialist view that «femininity» and marriage are protective factors. SP patterns illustrate and complement the explanatory concepts of SP. The inner world of suicidal people is fascinating and contributes to the evidence-based optimism in the «patient-centric» crisis care model.

**Disclosure of Interest:**

None Declared

